# Supplementary material for: Characterization of Aspartic Proteases from Paracoccidioides brasiliensis and Their Role in Fungal Thermo-Dimorphism
Source: J Fungi (Basel). 2023 Mar 19;9(3):375. doi: 10.3390/jof9030375 (PMC10053120; doi:10.3390/jof9030375)
Supplement: Supplementary file 1 [file jof-09-00375-s001.zip › jof-2140292-supplementary.pdf]

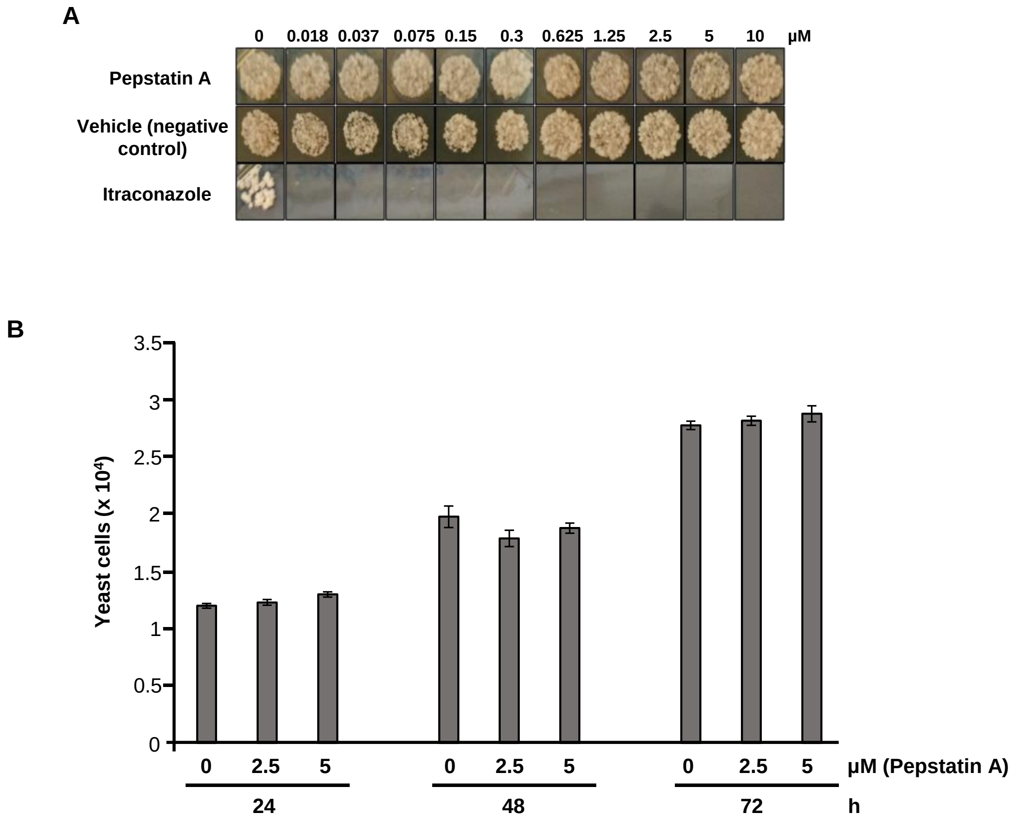

Supplementary Figure 1. Evaluation of the cytotoxicity of Pepstatin A in *P. brasiliensis*. (A) Yeast cells (1 x 10^4) were cultured in microplates containing RPMI medium and treated with Pepstatin A (0.018 - 10 µM), Methanol (vehicle) or Itraconazole (0.01 - 10 µM) (Positive Control). The culture was incubated for 7 days at 37°C and after that period 10 µL of each point was applied in YPDmod medium and incubated again for 5 days at 37°C. **(B)** Yest cells were treated with Pepstatin A (2.5 and 5 µM) and fungal growth was determined by counting in a Neubauer chamber. Representative data from three independent experiments.

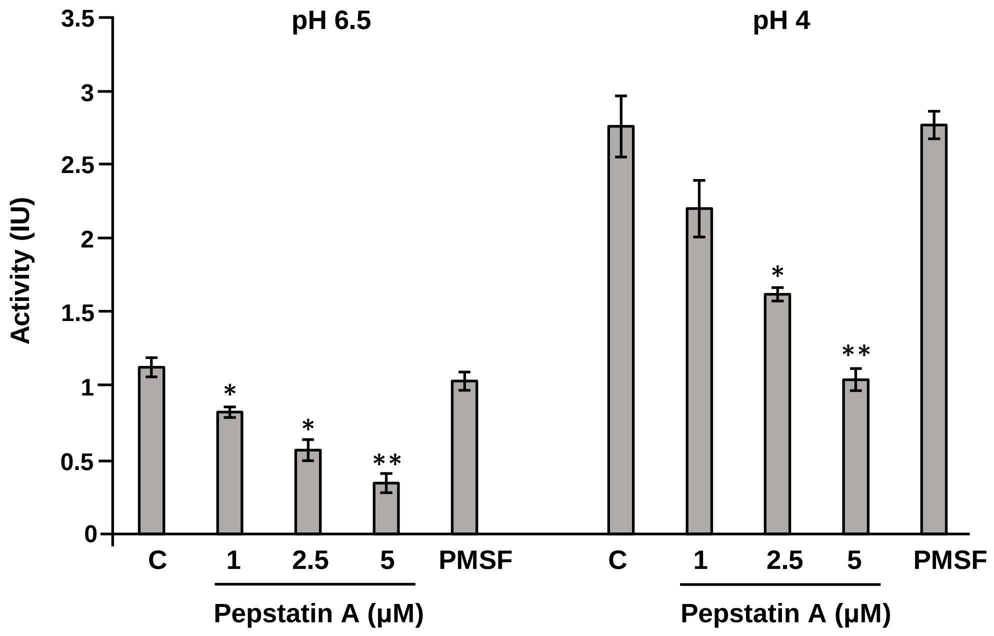

Suplementary Figure 2. Proteolytic activity profile of acid proteases from *P. brasiliensis*. Yeast cells were grown in YPDm with pH 6.5 or 4 for five days. Proteolytic activity was measured using 3 μg *P. brasiliensis* protein extract and 80 μL of 7.5 μM bovine serum albumin (BSA) in 0.1 M citric acid/sodium phosphate, pH 3.3, and incubated at 50°C. Samples were incubated in the presence or absence of Pepstatin A (1, 2.5 and 5 μM) or PMSF (100 μM). Then, 20 μL was removed after 30 min and added to 180 μL of Coomassie PlusTM protein assay reagent (Pierce) in a microtiter plate. Absorbance was read at 590 nm, and activity (IU) was calculated relative to BSA degradation using a standard curve. One IU is defined as degradation of 1 μmol of BSA $min^{-1}.ml^{-1}$. *$p \leq 0.05$ and **$p \leq 0.01$. The results shown are representative of two independent experiments.
